# Efficacy of oral ferric citrate hydrate treatment for anemia caused by niraparib: a case report

**DOI:** 10.1186/s13256-022-03666-3

**Published:** 2022-11-25

**Authors:** Hiroshi Kobayashi, Yuki Yamada

**Affiliations:** 1 Department of Obstetrics and Gynecology, Ms.Clinic MayOne, Kashihara, Nara 634-0813 Japan; 2 Department of Obstetrics and Gynecology, Nara Medical University, 840 Shijo-Cho, Kashihara, Nara 634-8522 Japan

**Keywords:** Anemia, Ferric citrate hydrate, Niraparib, Ovarian cancer

## Abstract

**Background:**

Maintenance therapy using poly(adenosine diphosphate-ribose)polymerase inhibitors may have adverse events, including hematological toxicity, and may limit therapeutic potential in patients with cancer. Niraparib-induced anemia negatively impacts one’s quality of life. Its amelioration by ferrous iron (for example, sodium ferrous citrate), folic acid, or vitamin B12 has not been supported. Oral ferric citrate hydrate increases circulating levels of iron and hepatic iron accumulation, improving renal anemia in patients with kidney failure receiving hemodialysis. The uptake of ferric iron is considered to be much higher than that of ferrous iron.

**Case presentation:**

The admitted patient was a 57-year-old Japanese woman with stage IIIB ovarian cancer who underwent primary debulking surgery and standard carboplatin–paclitaxel chemotherapy combined with bevacizumab, followed by niraparib (200 mg/day) maintenance therapy. The patient started oral SFC (100 mg/day) to treat niraparib-related anemia. However, she required two units of packed red blood cell transfusions three times within 3 months after starting niraparib treatment. The patient was diagnosed with niraparib-related anemia. The blood test results after 1 month from the start of niraparib treatment were as follows: red blood cells, 211 × 10^4^/μL; hemoglobin, 7.0 g/dL; hematocrit, 20.8%; reticulocyte, 0.2%; platelet count, 18.0 × 10^4^/μL. She was switched to oral ferric citrate hydrate with a dose of 500 mg per day and resumed niraparib treatment. She did not experience grade 3 niraparib-related hematological toxicity and achieved blood transfusion independence.

**Conclusions:**

Ferric citrate hydrate may be a safe, effective, and well-tolerated oral drug for treating patients with niraparib-related anemia.

## Background

Ovarian cancer is the leading cause of death from gynecological malignancy [1]. The first-line treatment for epithelial ovarian cancer (EOC) includes debulking surgery and platinum/taxane-based combination chemotherapy [2]. Poly(ADP-ribose)polymerase (PARP) inhibitors have changed the treatment strategies of EOC [3, 4]. Patients with EOC who have BRCA mutations and/or homologous recombination deficiencies (HRD) can benefit from PARP inhibitors on the basis of the synthetic lethality concept [4–7]. Therefore, for patients with advanced EOC who have BRCA mutations or HRD, PARP inhibitors such as niraparib are efficient as maintenance therapy. Adverse events associated with niraparib include hematologic abnormalities (anemia, thrombocytopenia, and leukopenia) and gastrointestinal symptoms (nausea and fatigue) [8]. Grade 3 or 4 anemia occurs in 25.3–28.7% of patients and is managed by dose reduction, interruption, discontinuation, or red blood cell (RBC) transfusion [9]. Anemia frequently occurs within 3 months after niraparib maintenance therapy [10]. Notably, whether iron, folic acid, or vitamin B12 supplementation ameliorates anemia caused by PARP inhibitors remains unclear.

Here, we report the case of a 57-year-old Japanese woman being treated with oral ferric citrate hydrate (FCH) for anemia associated with niraparib. Oral FCH may be a treatment option for niraparib-induced anemia.

## Case presentation

**Fig. 1:**
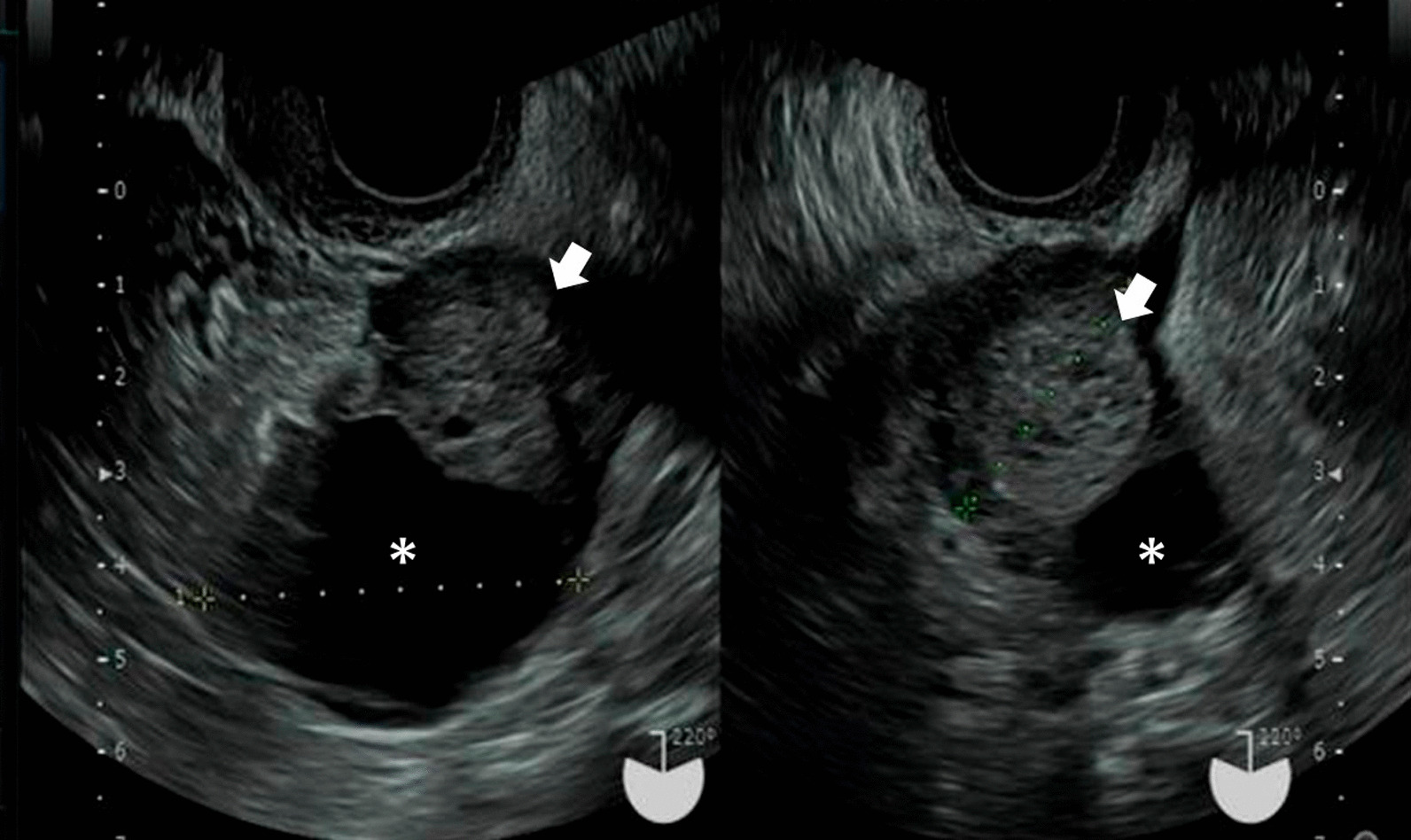
TVS of a cystic mass with solid component in the pelvis. Cystic and solid components measuring 60 × 35 mm in diameter localized at the left adnexa, suggestive of a lesion of the left ovary. *, a cystic mass with a maximum diameter of 4.8 cm. Arrow, a solid mass with a maximum diameter of 3.1 cm. *TVS* transvaginal ultrasound

**Fig. 2:**
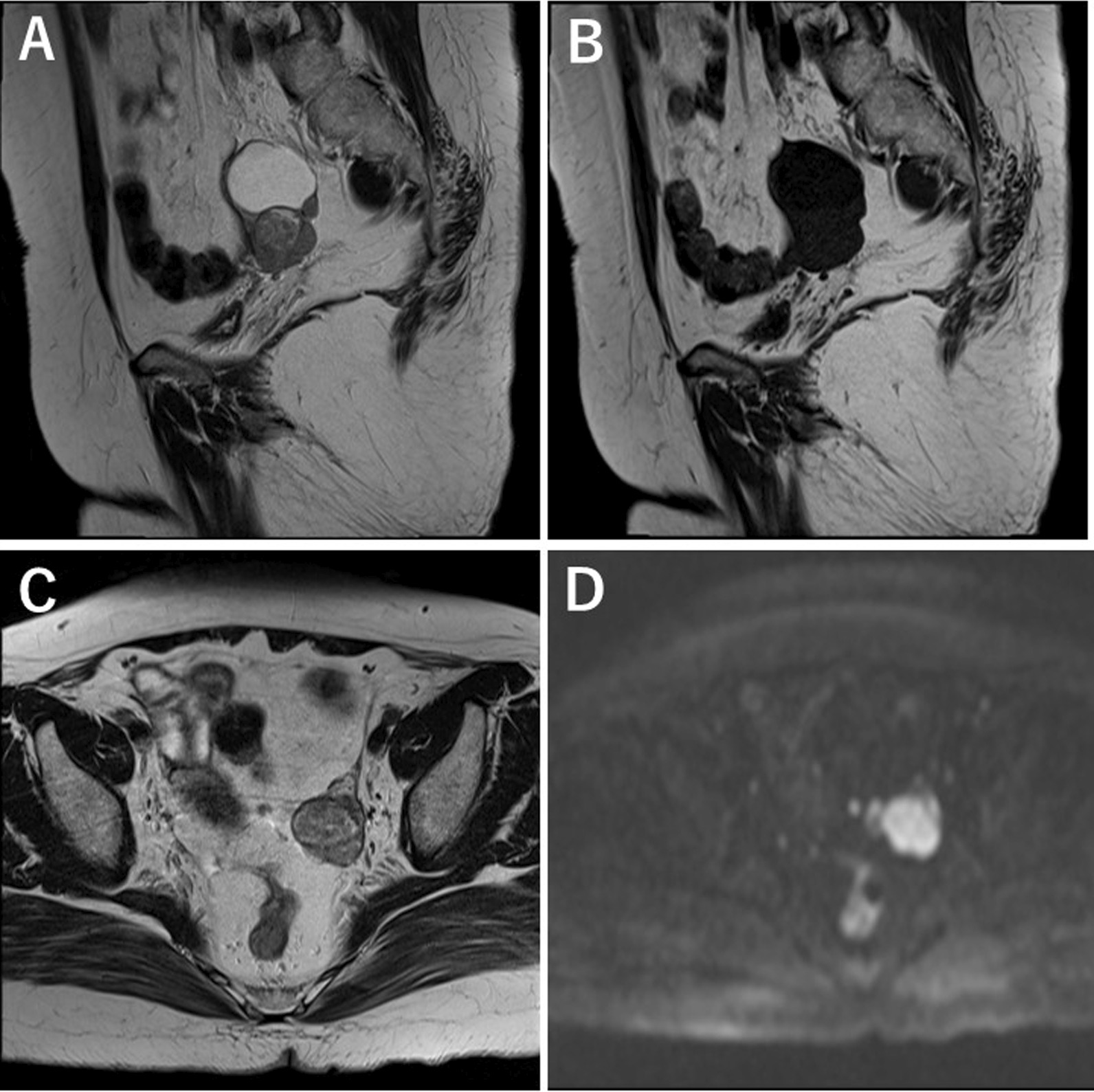
MRI of a mass in the pelvis. A 57 × 41 × 39 mm cystic mass with solid component originating in the left adnexa. **A** and **C** T2-weighted MRI; **B** T1-weighted MRI; **D** diffusion-weighted images. **A** and **B** MRI in sagittal planes. **C** and **D** MRI in axial planes. *MRI* magnetic resonance imaging

**Fig. 3:**
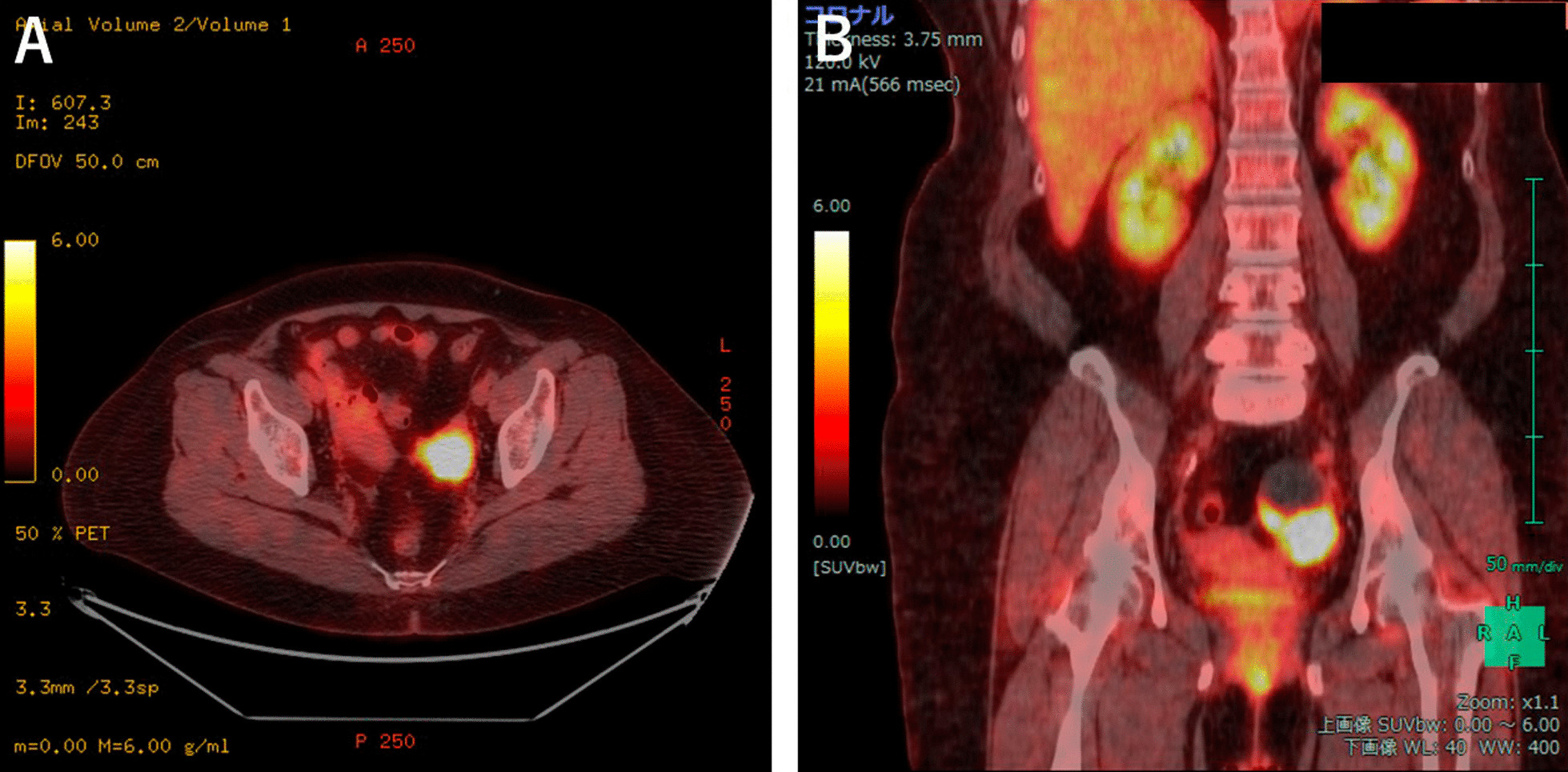
PET/CT scan of a mass in the pelvis. **A** PET/CT fusion image; and **B** coronal image. *PET/CT* positron emission tomography/computed tomography

**Fig. 4:**
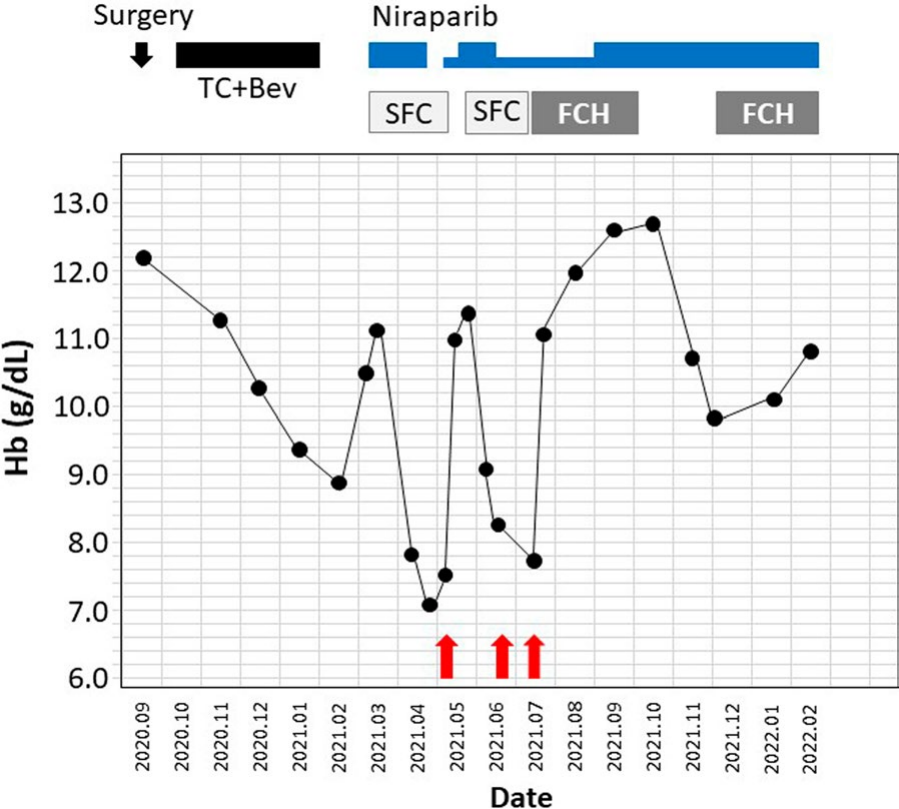
Effect of oral iron treatment on hemoglobin levels. Niraparib-related anemia was managed by dose interruption, dose reduction, or discontinuation. The patient was treated with oral SFC and blood transfusions for anemia. Thereafter, she began taking oral FCH depending on hemoglobin level. Hb, hemoglobin (g/dL); black arrow, surgery; red arrow, packed red blood cell transfusion; black square, chemotherapy consisting of paclitaxel (175 mg/m^2^; 3-hour infusion) and carboplatin [at a dose corresponding to an area under the curve (AUC) of 5 mg/mL/minute] combined with bevacizumab (15 mg/kg every 3 weeks); blue square, oral niraparib maintenance therapy at a bolus of 200 mg daily (wide blue square) and 100 mg daily (narrow blue square); *SFC* oral sodium ferrous citrate, *SCH* oral ferric citrate hydrate

A 57-year-old nulliparous Japanese woman visited our clinic for the first time, presenting with dull left lower abdominal pain lasting for a few weeks. Transvaginal ultrasound (TVS) showed a left unilocular ovarian cystic mass, measuring 60 mm, containing a solid component on the cul-de-sac of Douglas (Fig. 1). The patient was referred to the Department of Gynecology, Nara Medical University Hospital in September 2020 for the diagnosis of ovarian tumor. The patient was a housewife with normal social, emotional, and cognitive development and had normal environmental and employment history. There was no past medical, surgical, and medication history. She had never smoked or consumed alcohol. Neurological examinations were unremarkable. Evaluation of the patient’s family history revealed that her father died of lung cancer, all three of her paternal uncles succumbed to colon cancer, two paternal aunts died of gastric cancer and colon cancer, respectively, and one maternal uncle was treated for leukemia. In addition, her paternal grandfather died of gastric cancer. All three of her brothers are fine. Vital signs on admission showed a body temperature of 36.3 °C, blood pressure of 132/74 mm Hg, and respiratory rate of 20/minute. She was obese (weight 79 kg, height 158 cm, body mass index 31.6 kg/m^2^). A pelvic mass was palpated on the left side of the uterus during physical examination. Pelvic magnetic resonance imaging (MRI) showed a 57 × 41 × 39 mm cystic mass with solid component originating in the left adnexa. The cystic part is of liquid signal, the solid component had high signal intensity on T2-weighted imaging (T2WI), and had low signal intensity on T1-weighted imaging (T1WI), with hyperintensity on diffusion-weighted images (DWI) (Fig. 2). In addition, ^18^F-fluorodeoxyglucose (FDG) positron emission tomography/computed tomography (PET/CT) scan revealed increased FDG uptake in solid components of the tumor [maximum standardized uptake value (SUVmax): 19.9] (Fig. 3). No distant metastases were detected with staging CT and FDG-PET/CT. The patient had elevated serum levels of cancer antigen (CA) 125 (89 U/mL), but CA19-9 and carcinoembryonic antigen (CEA) were within the normal range. Imaging studies suspected ovarian malignancy, and she received primary debulking surgery. Following surgery, the pathological evaluation revealed the International Federation of Gynecology and Obstetrics (FIGO) stage IIIB high-grade serous ovarian cancer (pT3bN1M0). The patient underwent complete cytoreductive surgery. On postoperative day 27, the patient received the first cycle of combination chemotherapy comprising paclitaxel (175 mg/m^2^; 3-hour infusion) and carboplatin (at a dose corresponding to an area under the curve of 5 mg/mL/minute). The patient subsequently received five cycles of standard carboplatin–paclitaxel chemotherapy combined with bevacizumab (15 mg/kg every 3 weeks). Genetic analysis of peripheral blood DNA revealed that BRCA1 and BRCA2 gene mutations were absent, but tissue samples from a pretreatment tumor biopsy were positive for the homologous recombination deficiency (HRD) test (Myriad Genetics, Inc.). The patient received oral niraparib maintenance therapy at a bolus of 200 mg daily after first-line chemotherapy. Figure 4 and Table 1 show the laboratory data. Her RBC count, hemoglobin, white blood cell count, and platelet count were 297 × 10^4^/μL, 10.5 g/dL, 35 × 10^2^/μL, and 21.1 × 10^4^/μL, respectively, when she started niraparib therapy. Immediately after the start of niraparib treatment (that is, 6 months after surgery), sodium ferrous citrate (SFC) was orally administered to treat anemia. Forty days after the start of niraparib therapy, peripheral blood cell counts, hematocrit levels, platelet counts, white blood cell count, and neutrophil count decreased to 231 × 10^4^/μL, 7.8 g/dL, 9.6 × 10^4^/μL, 18 × 10^2^/μL, and 323/μL, respectively (Table 1). Niraparib therapy was discontinued, but hemoglobin levels were further reduced to 7.0 g/dL. The patient had transient neutropenia and thrombocytopenia. Blood examination showed macrocytic anemia [mean corpuscular volume, 99.1 fL (82–98 fL); mean corpuscular hemoglobin, 33.8 pg (26–32 pg/mL); and mean corpuscular hemoglobin concentration, 35.1 g/dL (31–34 g/dL)]. Furthermore, blood tests showed high serum ferritin levels 350 ng/mL (10–148 ng/mL) and reticulocyte 3.5% (0.5–2.0%), low serum total iron-binding capacity 260 μg/dL (263–457 μg/dL), and serum levels of iron 141 μg/dL (32–170 μg/dL), bilirubin 0.6 mg/dL (0.4–1.5 mg/dL), lactate dehydrogenase 190 U/L (120–225 U/L), folic acid 8.0 ng/mL (2.1–10.1 ng/mL), vitamin B12 401 pg/mL (180–914 pg/mL), and transferrin 211 mg/dL (190–320 mg/dL) were in the normal range. Chest radiography, electrocardiogram, abdominal ultrasound, and thorax, abdominal, and pelvic computerized tomography were normal. Therefore, the patient was diagnosed with niraparib-related anemia. Niraparib therapy was discontinued for 2 weeks, and two units of packed RBC were transfused. Thereafter, the patient resumed oral niraparib therapy at a bolus of 100 mg daily and then increased to 200 mg 2 weeks later, but immediately exhibited grade 3 anemia. The patients required two units of packed RBC transfusions twice to maintain serum hemoglobin levels above 10 g/dL. RBC transfusions, dose interruption, and dose reduction were used to manage grade 3 anemia. SFC was discontinued and changed to oral FCH after blood transfusion. She began taking oral FCH at a dose of 500 mg daily 10 months after her surgery. Niraparib therapy was maintained at a daily dose of 100 mg until the following month. After continuous administration of FCH, the peripheral blood cell counts recovered gradually with stable Hb counts fluctuating between 10.7 and 12.5 g/dL. One year after surgery, niraparib therapy is still maintained at a daily dose of 200 mg. Oral administration of FCH was discontinued because the hemoglobin level exceeded 12 g/dL. Then, the serum hemoglobin levels decreased again. However, our patient no longer required blood transfusions and Hb levels stabilized at around 10–11 g/dL. She resumed oral FCH therapy after 8 weeks of pause, showing efficacy again. The follow-up is ongoing, and she has never been transfused. Table 1 presents laboratory findings in this patient before and after treatment with FCH. She has survived disease-free for 25 months after initial surgery. There is no sign of radiological recurrence with a normal serum CA125 level until now. She reached a total dose of approximately 90,000 mg and 135,000 mg of niraparib and FCH, respectively. We obtained the patient’s informed consent to publish this case report.

**Table 1:** Laboratory findings before and after treatment with ferric citrate hydrate (FCH)

	Before oral administration of FCH	After oral administration of FCH	Normal reference range
Peripheral blood cell counts, × 10^4^/μL	231	324	386–492
Hemoglobin	7.8	11.6	11.6–14.8
Hematocrit levels, g/dL	22.8	32.4	35.1–44.4
Platelet counts, × 10^4^/μL	9.6	19.7	15.8–34.8
White blood cell count, × 10^2^/μL	18	32	33–86
Neutrophil count, /μL	323	2394	
CA125, U/mL	22	7	< 35
Serum ferritin level, ng/mL	350	361	10–148
Reticulocyte, %	3.5	3.1	0.5–2.0
Total iron-binding capacity, μg/dL	260		263–457
Iron, μg/dL	141		32–170
Bilirubin, mg/dL	0.6	0.5	0.4–1.5
Folic acid, ng/mL	8.0		2.1–10.1
Vitamin B12, pg/mL	401		180–914
Transferrin, mg/dL	211		190–320
Aspartate aminotransferase (AST), U/L	19	32	13–30
Alanine aminotransferase (ALT), U/L	20	46	7–23
Lactate dehydrogenase (LDH), U/L	190	185	120–225
Alkaline phosphatase (ALP), U/L	111	147	38–113
Blood urea nitrogen (BUN), mg/dL	15	12	8–20
Creatinine	0.81	0.77	0.46–0.79
Urine pH	6.5	6.5	
Urine protein, mg/dL	300	30	
Urine glucose, mg/dL	–	–	
Urine ketone bodies	–	–	
Urinary occult blood	–	–	
Urine urobilinogen, EU/dL	0.1	0.1	
Urine bilirubin	–	–	

## Discussion and conclusions

We describe herein an EOC patient with niraparib-related anemia who has been successfully treated with oral FCH. Niraparib is a PARP inhibitor that yields synthetic lethality in patients with EOC [4–7]. A preferential benefit from niraparib was seen in patients with germline BRCA1/2 mutations or the HRD test-positive subgroup [3–7]. However, patients’ adverse events, such as hematologic abnormalities (anemia and leukopenia) and gastrointestinal symptoms (nausea and fatigue) during niraparib treatment, affect their quality of life [8, 9]. Adverse effects greater than grade 2, such as severe anemia, are manageable with dose interruptions or reductions plus supportive care [11]. Blood transfusions as supportive care may be required for symptomatic anemia [12]. To date, there are no reported treatments for symptomatic anemia other than blood transfusions. In our case, grade 3 neutropenia was reversible upon cessation of niraparib or reduction of the dose, while grade 3 or higher anemia was managed by frequent RBC transfusions. We found for the first time that oral SFC treatment was ineffective, but oral FCH improved the treatment outcome of anemia caused by PARP inhibitors. In addition, she did not show any adverse events due to oral FCH.

FCH is an oral phosphate binder that improves hyperphosphatemia and iron deficiency in patients with chronic kidney disease [13]. Ferric iron is much more readily absorbed by renal tubular epithelial cells than ferrous iron [14]. Oral FCH is clinically useful for treating renal anemia in patients undergoing hemodialysis [15]. Therefore, effective erythropoiesis may depend on the quick uptake of iron in the ferric form. Her blood reticulocyte count 3.5% (0.5–2.0%) was slightly elevated, but erythropoietin 11.8 mIU/mL (4.2–23.7 mIU/mL), LDH, and bilirubin levels were normal, eliminating the diagnosis of renal and hemolytic anemias. In addition, she cannot be diagnosed with iron deficiency anemia due to macrocytic anemia and high ferritin levels of 350 ng/mL (10–148 ng/mL). The reticulocyte count did not change before or after the FCH treatment. Therefore, there is no evidence that ferric citrate enhances the erythropoietic activity of the bone marrow. To our knowledge, this is the first case report of the efficacy of oral FCH treatment for niraparib-related anemia. Oral FCH may be effective in treating niraparib-related anemia owing to its even lower risk of nausea and vomiting than oral SFC. Therefore, ferric iron may be recommended instead of ferrous iron when starting PARP inhibitor treatment. The lessons learned from the presented case are that oral FCH may be considered an alternative first choice option for the treatment of niraparib-induced severe anemia.

The number of patients with ovarian cancer shows an increasing trend in Japan. Delayed referrals can be fatal for patients with cancer. Efficient referral networks and emergency transportation systems have been established in Japan under its universal health insurance system. These systems, in principle, do not differ between urban and rural areas. In addition, the social health insurance programs for urban and rural residents are the same, and no difference exists in out-of-pocket medical expenditure and reimbursement. The drug price of FCH in Japan is as low as 158 yen per day. FCH is also marketed in the USA under the trade name Auryxia.

In conclusion, oral FCH treatment may be effective for treating niraparib-related anemia. Further research is needed to elucidate the efficacy of oral FCH supplements for niraparib-related anemia and its underlying mechanism.

## Data Availability

All data generated or analyzed during this study are included in this published article.

## Abbreviations

EOC: Epithelial ovarian cancer
FCH: Ferric citrate hydrate
HRD: Homologous recombination deficiencies
PARP: Poly (ADP-ribose) polymerase
RBC: Red blood cell
SFC: Sodium ferrous citrate
